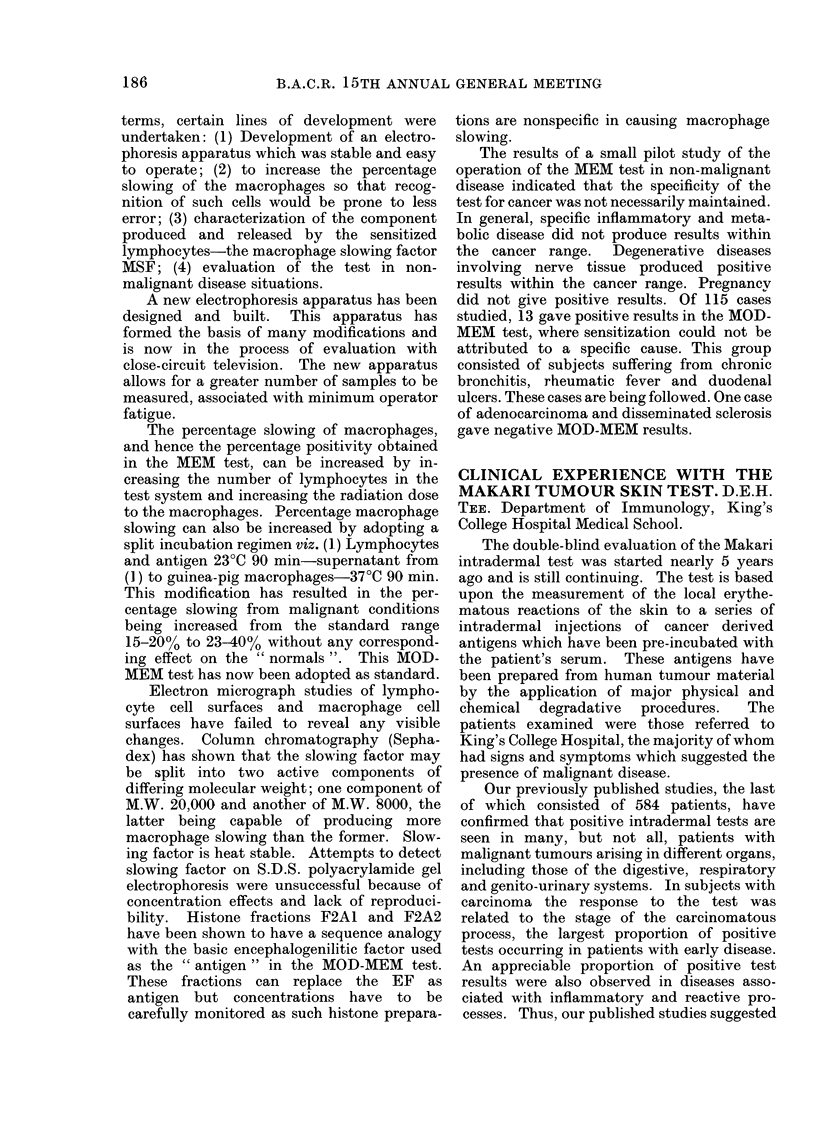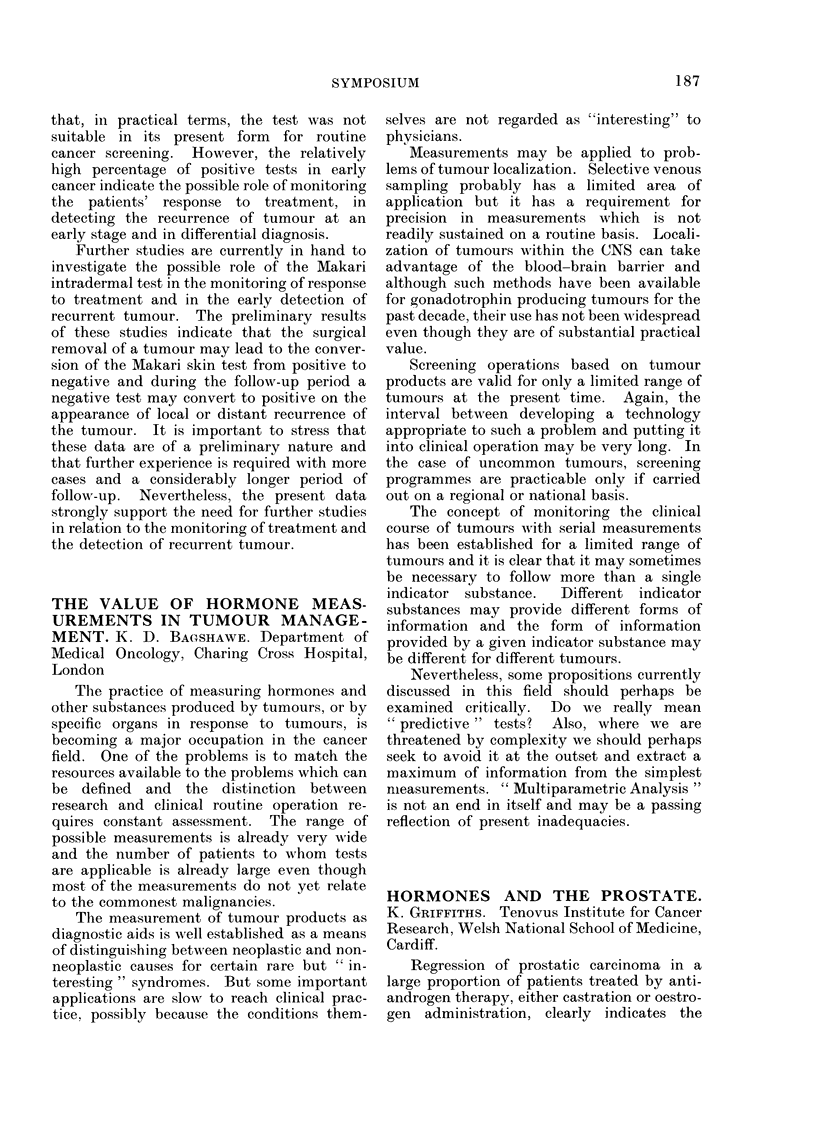# Proceedings: Clinical experience with the Makari tumour skin test.

**DOI:** 10.1038/bjc.1974.174

**Published:** 1974-08

**Authors:** D. E. Tee


### CLINICAL EXPERIENCE WITH THE MAKARI TUMOUR SKIN TEST.

D.E.H. Tee. Department of Immunology, King's College Hospital Medical School.

The double-blind evaluation of the Makari intradermal test was started nearly 5 years ago and is still continuing. The test is based upon the measurement of the local erythematous reactions of the skin to a series of intradermal injections of cancer derived antigens which have been pre-incubated with the patient's serum. These antigens have been prepared from human tumour material by the application of major physical and chemical degradative procedures. The patients examined were those referred to King's College Hospital, the majority of whom had signs and symptoms which suggested the presence of malignant disease.

Our previously published studies, the last of which consisted of 584 patients, have confirmed that positive intradermal tests are seen in many, but not all, patients with malignant tumours arising in different organs, including those of the digestive, respiratory and genito-urinary systems. In subjects with carcinoma the response to the test was related to the stage of the carcinomatous process, the largest proportion of positive tests occurring in patients with early disease. An appreciable proportion of positive test results were also observed in diseases associated with inflammatory and reactive processes. Thus, our published studies suggested that, in practical terms, the test was not suitable in its present form for routine cancer screening. However, the relatively high percentage of positive tests in early cancer indicate the possible role of monitoring the patients' response to treatment, in detecting the recurrence of tumour at an early stage and in differential diagnosis.

Further studies are currently in hand to investigate the possible role of the Makari intradermal test in the monitoring of response to treatment and in the early detection of recurrent tumour. The preliminary results of these studies indicate that the surgical removal of a tumour may lead to the conversion of the Makari skin test from positive to negative and during the follow-up period a negative test may convert to positive on the appearance of local or distant recurrence of the tumour. It is important to stress that these data are of a preliminary nature and that further experience is required with more cases and a considerably longer period of follow-up. Nevertheless, the present data strongly support the need for further studies in relation to the monitoring of treatment and the detection of recurrent tumour.